# Cardiac Glycoside Ouabain Exerts Anticancer Activity *via* Downregulation of STAT3

**DOI:** 10.3389/fonc.2021.684316

**Published:** 2021-06-30

**Authors:** Jie Du, Lijun Jiang, Fuqiang Chen, Huantao Hu, Meijuan Zhou

**Affiliations:** ^1^ Jiangmen Central Hospital, Affiliated Jiangmen Hospital of Sun Yat-sen University, Jiangmen, China; ^2^ Department of Radiation Medicine, Guangdong Provincial Key Laboratory of Tropical Disease Research, School of Public Health, Southern Medical University, Guangzhou, China; ^3^ The First School of Clinical Medicine, Southern Medical University, Guangzhou, China

**Keywords:** ouabain, STAT3, protein synthesis, eIF4E, 4EBP1

## Abstract

Cardiac glycosides are plant-derived steroid-like compounds which have been used for the treatment of cardiovascular diseases. Ouabain, a cardiotonic steroid and specific Na^+^/K^+^-ATPase inhibitor, has been rediscovered for its potential use in the treatment of cancer. However, the cellular targets and anticancer mechanism of ouabain in various cancers remain largely unexplored. In this study, we confirmed the cytotoxic effects of ouabain on several cancer cell lines. Further examination revealed the increase of apoptosis, intracellular ROS generation and DNA double-strand breaks induced by ouabain treatment. Besides, ouabain effectively suppressed STAT3 expression as well as phosphorylation in addition to block STAT3-mediated transcription and downstream target proteins. Interestingly, these inhibitory activities seemed to be independent of the Na^+^/K^+^-ATPase. Furthermore, we found that ouabain inhibited protein synthesis through regulation of the eukaryotic initiation factor 4E (eIF4E) and eIF4E binding protein 1 (4EBP1). Taken together, our study provided a novel molecular insight of anticancer activities of ouabain in human cancer cells, which could raise the hope of using cardiac glycosides for cancer therapeutics more rational.

## Introduction

Ouabain belongs to a large family of plant-derived steroid-like compounds known as cardiac glycosides which have been used for the treatment of congestive heart failure for a long time (1). The pharmacological action of cardiac glycosides is based on the inhibition of Na^+^/K^+^-ATPase on the cardiomyocyte membrane, causing an increase in the levels of intracellular sodium ions which further increase the levels of calcium ions and exert positive inotropic effects (2). The first evidence of cytotoxic effects of cardiac glycosides on human cancer cells *in vitro* was reported in 1967 (3). In the 1980s, it was reported a considerable reduction of recurrence rate of breast cancer in the patients who were treated with cardiac glycosides for heart failure (4). A 20-year follow-up study revealed that breast cancer patients taking cardiac glycosides had a lower mortality rate compared with the control group (5). These encouraging data promoted the repurposing of cardiac glycosides for cancer treatment and numerous reports have affirmed the antineoplastic activity of many kinds of cardiac glycosides, such as ouabain, digoxin and lanatoside C (6).

**Figure 1:**
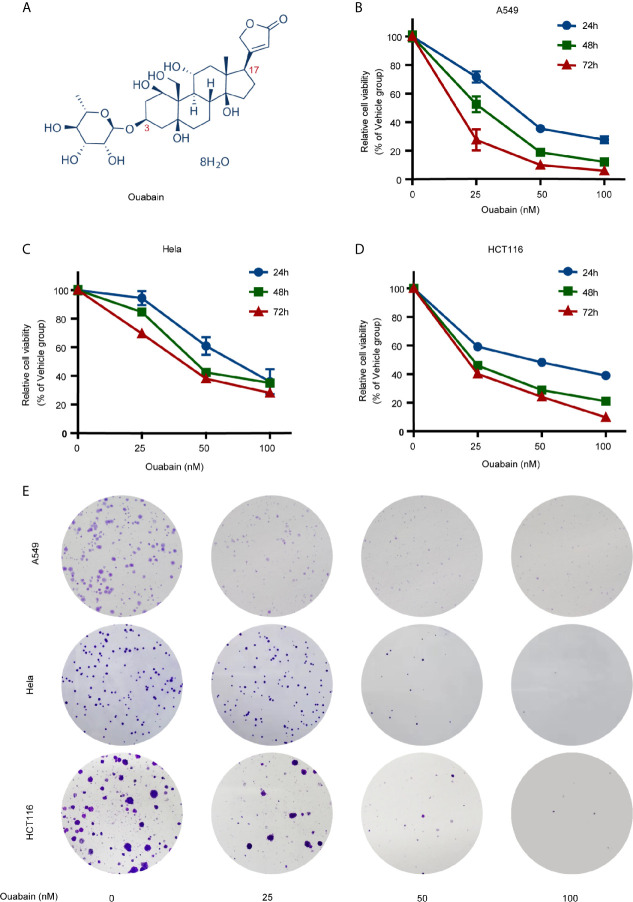
Effects of ouabain on the viability and clonogenicity of various cancer cells. **(A)** Chemical structure of ouabain. **(B–D)** A549, Hela and HCT116 cells were treated with ouabain (0-100 nM) for 24, 48 or 72 hours and cell viability was determined by CCK-8 assay. **(E)** A549, Hela and HCT116 cells were treated with different doses of ouabain (0, 25, 50, 100 nM) for 24 hours and cell clonogenicity were measured by colony formation assay.

The structure of ouabain has a steroid core with a five-membered unsaturated butyrolactone ring at position 17 and a rhamnose residue at position 3 (**Figure 1A**) (7). Increasing literatures have demonstrated the antiproliferative effects of ouabain on various cancer cells including breast cancer (8), lung cancer (9), prostate cancer (10), colon cancer (11) and leukemia (12). Although the detailed mechanisms have not been fully elucidated, ouabain has been shown to exert antitumor effects manifested in the activation of various modes of cell death, including apoptosis, autophagy and immunogenic cell death (13–15). A better understanding of the cytotoxic effects of ouabain on cancer cells would pave the way for their clinical application and rational design of combination treatment.

Signal transducer and activator of transcription 3 (STAT3) is a DNA-binding protein playing dual roles as signal transducers and transcription factors. Recently, ouabain has been shown to suppress the IL-6/STAT3 signaling but promote the secretion of IL-6 and other cytokines in the cultured human skeletal muscle cells (16). In contrast to the transient nature of STAT3 activation in normal cells, the constitutive or aberrant activation of STAT3 signaling has been found in a wide-range of human cancers in which STAT3 promotes cell proliferation, anti-apoptosis, metastasis, angiogenesis, immune evasion and resistance to chemotherapy and radiotherapy (17–19). However, only limited data regarding the effect and molecular mechanism of action of ouabain on STAT3 in human cancer cells is available. More details involved in ouabain inhibition of STAT3 are worthy of assessment.

In this study, we confirmed the cytotoxic effects of ouabain on several cancer cell lines and found that ouabain decreased the expression of STAT3 and thus prevented the activation of STAT3 signaling. Interestingly, these effects seemed to be independent of the Na^+^/K^+^-ATPase. Further experiments suggested that the inhibitory effects of ouabain on STAT3 might be through attenuating the translation of STAT3 protein.

## Materials and Methods

### Drugs and Reagents

Ouabain (purity ≥ 99%), digoxin and lanatoside C were purchased from MedChemExpress (Monmouth Junction, NJ, USA).MG132 was purchased from Selleck Chemicals (Houston, TX, USA). Cycloheximide (CHX) was purchased from Sigma (St. Louis, MO, USA). All drugs were dissolved in dimethyl sulfoxide (DMSO) to obtain a stocking solution and stored at -20°C. Recombinant human interferon-γ (IFN-γ) was purchased from Beyotime Biotechnology (Shanghai, China) and reconstituted in sterile distilled water containing 0.1% bovine serum albumin.

### Cell Culture

Human non-small-cell lung cancer cell line A549 and H460, colorectal carcinoma cell line HCT116, pancreatic cancer cell line PANC1 and cervical cancer cell line Hela were obtained from American Type Culture Collection. Cells were cultured in Dulbecco’s modified Eagle’s medium (H460, PANC1 and HeLa cells) or Ham’s F12K medium (A549 cells) or McCoy’s 5A medium (HCT116 cells) supplemented with 10% fetal bovine serum, 100 μg/ml streptomycin and 100 U/ml penicillin at 37°C under a humidified atmosphere containing 5% CO_2_.

### Cell Viability Assay

Cells were seeded in 96-well microplates at 5,000 cells/well and cultured overnight to allow cells to adhere. Then cells were treated with different concentrations of ouabain or DMSO. After treatment for 24, 48 or 72 hours, cell viability was evaluated by using Cell Counting Kit-8 (CCK-8) (Yeasen, Shanghai, China) referring to the manufacture’s protocol. The OD values at 450 nm were detected after incubation for 2 hours.

### Clonogenic Survival Assay

Cells were counted and plated into 6-well plates at 250 cells/well in triplicate. The next day, cells were incubated with different concentrations of ouabain for 24 hours. Then the cells were replaced with fresh medium and further cultured for 7-14 days. Cell colonies were fixed with methanol and stained with 1% crystal violet.

### Flow Cytometry Analysis

Cells were seeded in 6-well plates. After incubation with ouabain for 24 or 48 hours, cells were harvested and washed twice with phosphate-buffered saline (PBS). Apoptosis cells were quantified using Annexin V-FITC/PI double staining with an Apoptosis Detection Kit (BD Biosciences, San Jose, CA, USA). The stained cells were analyzed by BD LSRFFortessa X-20 Flow Cytometer.

### Western Blotting

Total cell lysates were prepared using cell lysis buffer containing protease inhibitor cocktail (Beyotime Biotechnology, Shanghai, China). Nuclear and cytoplasmic proteins were extracted using a commercial kit (Beyotime Biotechnology, Shanghai, China). Equal amounts of total protein were separated by SDS-PAGE, and electrically transferred to PVDF membranes (Millipore, Bedford, MA, USA). After blocking with 5% non-fat milk, the membranes were incubated with primary antibody for STAT3, phospho-STAT3^Tyr705^, mTOR, phospho-mTOR^Ser2448^, eIF4E, eIF4G, phospho-4EBP1^Thr70^, Caspase3, PARP, γ-H2AX^Ser139^, Lamin B, c-Myc, Survivin, Bcl-2 (Cell Signaling Technology, Danvers, MA, USA) and β-actin, 4EBP1 (Abcam, Cambridge, UK). Next, the membranes were washed with Tris-buffered saline with Tween 20 (TBST) buffer 3 times and incubated with horseradish peroxidase-conjugated second antibody. Immunoblotting signals were detected using an enhanced chemiluminescence method.

### Measurement of Intracellular Reactive Oxygen Species (ROS)

After incubation with ouabain for 24 hours, cells were harvested and incubated with 2’,7’-dichlorofluorescein diacetate (DCFH-DA) at 37°C for 20 min. After washing with serum-free DMEM three times, the cell pellets were gently resuspended in PBS. DCF-fluorescence intensity of each sample was analyzed by flow cytometry.

### Immunofluorescence

Hela cells were grown on coverslips and treated with vehicle or ouabain for 24 hours and fixed with 4% paraformaldehyde in the presence of 0.2% Triton X-100 for 20 min. Primary antibody against STAT3 or γ-H2AX (Cell Signaling Technology, Danvers, MA, USA) was diluted 1:500 to immunostain the cells at 4°C overnight. After washing, cells were further incubated with Alexa Fluor 555-labeled goat anti-mouse IgG (Invitrogen, CA, USA). A total of 100 randomly selected cells were analyzed for each group.

### siRNA Transfection

SiRNA duplexes against human Na^+^/K^+^-ATPase α1 subunit (siRNA-1: 5’-GATTCGAAATGGTGAGAAA-3’, siRNA-2: 5’-GTCGTCTGATCTTTGATAA-3’, siRNA-3: 5’-GAATTTCCCTATCGATAAT-3’) and control scrambled siRNA were synthesized by RiboBio (Guangzhou, China). The siRNA transfection was conducted using lipofectamine 3000 (Invitrogen, CA, USA) following the manufacturer’s protocol.

### Reverse Transcription-Polymerase Chain Reaction (RT-PCR)

Total RNA was extracted using TRIzol Reagent (Invitrogen, CA, USA). Reverse transcription was performed by PrimeScript RT Reagent Kit (Takara, Dalian, China) to obtain cDNA and mRNA analysis was performed by UltraSYBR Mixture (CWBio, Beijing, China). For semi-quantitative PCR, reverse transcription was carried out using PrimeScript^®^ RT-PCR kit (Takara, Dalian, China). PCR products were separated on a 2% agarose gel, stained with ethidium bromide. The primer sequences for PCR were as follows: STAT3, forward 5′-GGAGGAGTTGCAGCAAAAAG-3′, reverse 5′-TGTGTTTGTGCCCAGAATGT-3′; Na^+^/K^+^-ATPase α1 subunit, forward 5′-GCTGCTCTGTGCTTTTCTCTCT-3′, reverse 5′-CTGAAACAGCTGCAGGCTCATA-3′; GAPDH, forward 5′- GGATATTGTTGCCATCAATGACC-3′; reverse 5′- AGCCTTCTCCTGGTGAAGA-3′.

### Immunoprecipitation

For immunoprecipitation, cells were lysed on ice using RIPA buffer and then centrifuged at 12,000 rpm for 15 min. Subsequently, the cell lysates were subjected to immunoprecipitation using antibody-conjugated Protein A/G-Sepharose beads (Millipore, MA, USA), which were incubated overnight at 4°C. Immune complexes were extensively washed for three times and analyzed by Western blotting with specific antibodies.

### Statistical Analysis

Data were expressed as mean ± standard deviation (SD). Analysis was performed by Student’s t-test or one-way ANOVA following multiple comparisons using SPSS 20.0 software (SPSS Inc., Chicago, IL). Results with a *P*-value less than 0.05 were considered statistically significant.

## Results

### Cytotoxic Activity of Ouabain on Cancer Cells

A panel of human cancer cell lines was treated with ouabain at different concentrations for different durations. CCK-8 assay revealed that ouabain treatment impaired cell viability in a dose- and time-dependent manner (**Figures 1B–D**, **S1A**). Though the sensitivity of different cell lines to ouabain varied, nanomolar concentration of ouabain was capable to significantly decrease the cell viability of all these cell lines.

In the case of A549 cells, cell survival was reduced at 24, 48, and 72 h, respectively, to 71.7%, 52.6%, and 27.6% by 25 nM; to 35.6%, 18.9%, and 10% by 50 nM; to 27.8%, 12.3%, and 6.1% by 100 nM, compared to the vehicle control. Hela cell survival was reduced at 24, 48, and 72 h, respectively, to 94.4%, 84.7%, and 69.5% by 25 nM; to 60.9%, 42.4%, and 38% by 50 nM; to 35.9%, 35%, and 28% by 100 nM, compared to the vehicle control. HCT116 cell survival was reduced at 24, 48, and 72 h, respectively, to 59.2%, 46%, and 40.3% by 25 nM; to 48.3%, 28.9%, and 24.1% by 50 nM; to 39%, 21%, and 9.9% by 100 nM, compared to the vehicle control. The IC50 values of ouabain ranged from 10.44 nM (H460 cells) to 42.36 nM (PANC1 cells) when the cells were incubated with ouabain for 72 hours. Next, we evaluated the effects of ouabain on colony formation ability of A549, Hela and HCT116 cells. As shown in **Figure 1E**, incubation with 50 nM or 100 nM of ouabain for 24 hours greatly decreased the number of colonies (**Figure 1E**). All these findings suggested that ouabain exerted various growth inhibitory activities against all the cell lines tested.

### Induction of Apoptosis of Ouabain in Cancer Cells

**Figure 2:**
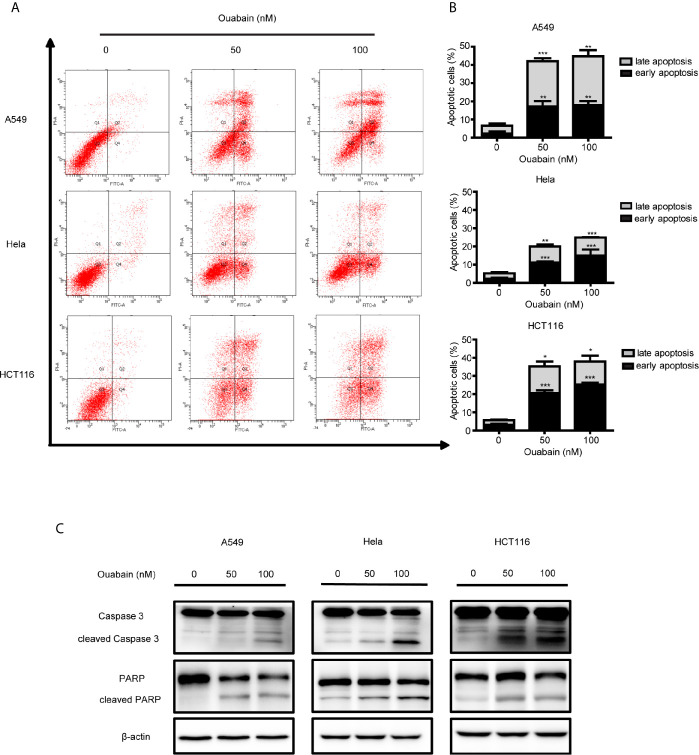
Ouabain induced apoptosis of cancer cells. **(A, B)** A549, Hela and HCT116 cells were treated with indicated dose of ouabain (0, 50, 100 nM) for 48 hours, apoptosis was tested by flow cytometric analysis with Annexin V-FITC/PI staining. **(C)** The expressions of PARP, cl-PARP, Caspase3, cl-Caspase3 were analyzed by Western blotting. β-actin was used as a loading control. ^*^ *P* < 0.05; ^**^ *P* < 0.01; ^***^ *P* < 0.001 *versus* control group, n = 3.

To determine whether ouabain-mediated cell death was due to the induction of apoptosis, we measured apoptosis with V-FITC/PI staining in the presence and absence of ouabain treatment. A549 cells, Hela cells and HCT116 cells were incubated with ouabain for 24 or 48 hours, and the apoptotic cells were quantified by flow cytometry. As shown in **Figures S1B, 1C**, ouabain induced apoptosis in a dose-dependent manner in A549, Hela and HCT116 cells. Flow cytometric analysis showed that ouabain treatment for 24 hours led to apoptotic rates from 5.73 ± 0.61% in the control group to 16.00 ± 1.3% (50 nM) and 27.77 ± 0.31% (100 nM) in A549 cells; from 4.43 ± 0.42% in the control group to 7.57 ± 0.12% (50 nM) and 13.87 ± 1.63% (100 nM) in Hela cells and from 5.73 ± 0.15% in the control group to 13.10 ± 0.17% (50 nM) and 18.30 ± 2.52% (100 nM) in HCT116 cells, respectively. After treatment with ouabain for 48 hours, the apoptotic rates were 42.03 ± 3.04% (50 nM), 44.68 ± 4.30% (100 nM) in A549 cells, 20.03 ± 5.18% (50 nM), 24.90 ± 3.21% (100 nM) in Hela cells and 35.28 ± 3.55% (50 nM), 37.75 ± 4.30% (100 nM) in HCT116 cells (**Figures 2A, B**). To further confirm these outcomes, we explored the hallmarks of apoptotic cell death. Western blot analysis showed the increased expression of cleaved Caspase3 and PARP in these three cell lines treated with ouabain for 48 hours (**Figure 2C**).

### Ouabain Increased ROS Production and Generated DNA Double-Strand Breaks

**Figure 3:**
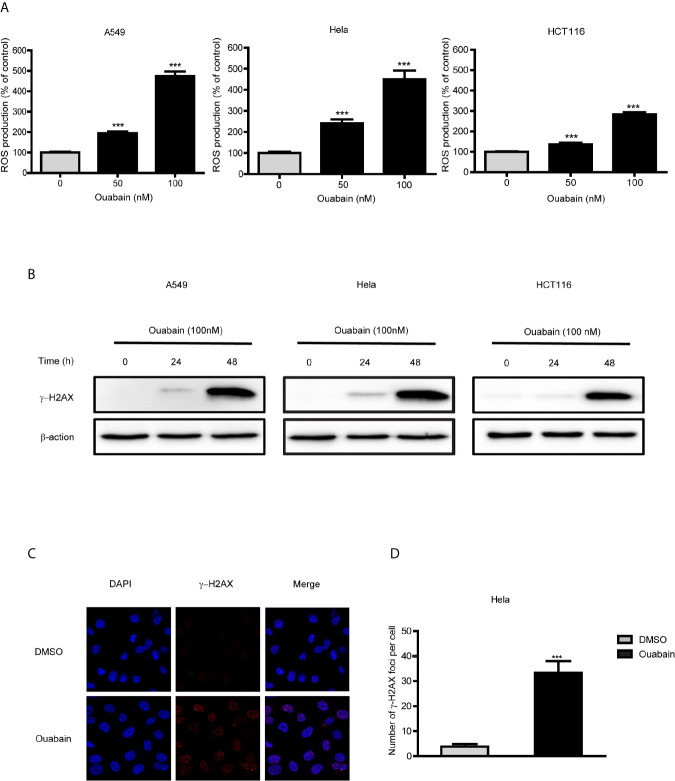
Ouabain increased ROS production and generated DNA double-strand breaks. **(A)** A549, Hela and HCT116 cells were treated with different concentrations of ouabain for 24 hours, and ROS generation was determined by staining the cells with DCFH-DA. **(B)** Cells were treated with ouabain (0, 50, 100 nM) for 24 or 48 hours, and expression of γ-H2AX were measured by Western blotting. **(C)** Hela cells were immunostained for γ-H2AX foci (red) after exposed to ouabain for 24 hours. **(D)** The number of γ-H2AX foci per cell. ^***^ *P* < 0.001 *versus* control group, n = 3.

Intracellular ROS generation by ouabain treatment in cancer cells was monitored by flow cytometry analysis. As shown in **Figure 3A**, ouabain treatment for 24 hours significantly increased ROS production to 195.3%, 240.8% and 162.0% compared to controls in A549, Hela and HCT116 cells, respectively (50nM); to 474.3%, 450.7% and 299.7% compared to controls in A549, Hela and HCT116 cells, respectively (100nM).

As highly unstable and reactive molecules, high levels of ROS could generate various DNA lesions. We performed Western blot analysis and immunofluorescence to detect the expression of γ-H2AX and γ-H2AX foci formation. The results showed that the expression of γ-H2AX significantly increased at 48 hours after treatment with ouabain in A549, Hela and HCT116 cells (**Figure 3B**). Additionally, γ-H2AX foci were significantly increased at 24 hours after treatment of ouabain (**Figures 3C, D**).

### Ouabain Downregulated the Expression of STAT3

**Figure 4:**
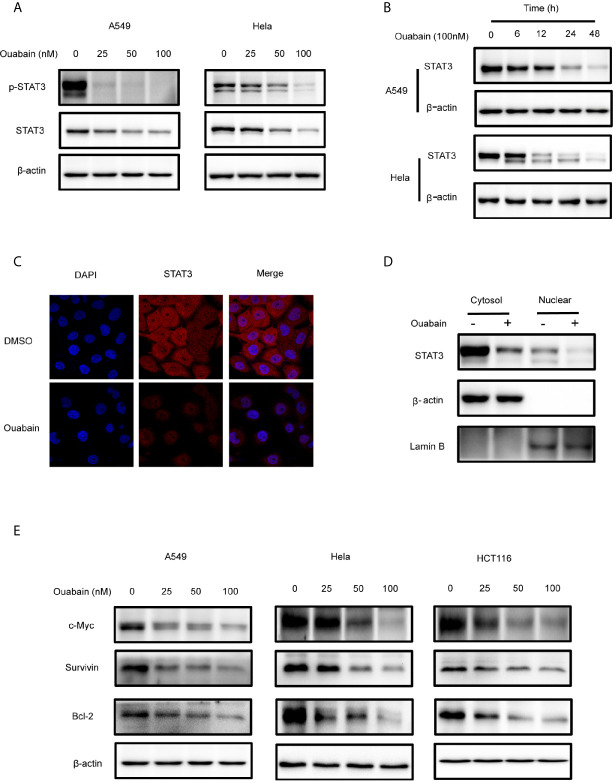
Ouabain downregulated the expression of STAT3. **(A, B, E)** Cells were incubated with ouabain at the indicated concentrations and time. The expressions of STAT3, p-STAT3, c-Myc, Survivin, Bcl-2 were determined by Western blotting using specific antibodies. **(C)** Hela cells treated with vehicle or 100 nM ouabain for 24 hours were analyzed by immunofluorescence. **(D)** Cytoplasmic and nuclear extracts from Hela cells treated with ouabain were analyzed by Western blotting using anti-STAT3.

Since the overexpression or persistent activation of STAT3 plays a critical role in the malignant progression in cancer cells, we then examined the effects of ouabain on STAT3 in the A549, Hela, HCT116 and PANC1 cells. Both concentration- and time-dependent decreases of STAT3 expression were observed in these cell lines (**Figures 4A, B**, **S2A**). The expression of p-STAT3 was also downregulated (**Figures 4A**, **S2A**). Immunofluorescent staining confirmed the reduced STAT3 levels in Hela cells treated with 100 nM ouabain for 24 hours (**Figure 4C**). Furthermore, subcellular fraction analysis revealed the reduction of STAT3 expression in both nucleus and cytoplasm (**Figure 4D**). It has been demonstrated that IFN-γ activates STAT3 phosphorylation and STAT3-dependent transcription. To further study the roles of ouabain on STAT3 inhibition, we investigated its effects on IFN-γ-dependent activation of STAT3. As shown in **Figure S2B**, ouabain treatment blocked the phosphorylation of STAT3 induced by IFN-γ. In addition, diminish of STAT3 expression by ouabain also reduced the expression of downstream target genes, such as c-Myc, Survivin and Bcl-2, which are involved in regulation of cell proliferation and apoptosis (**Figure 4E**).

### Downregulation of STAT3 by Ouabain Was Independent of Na^+^/K^+^-ATPase

**Figure 5:**
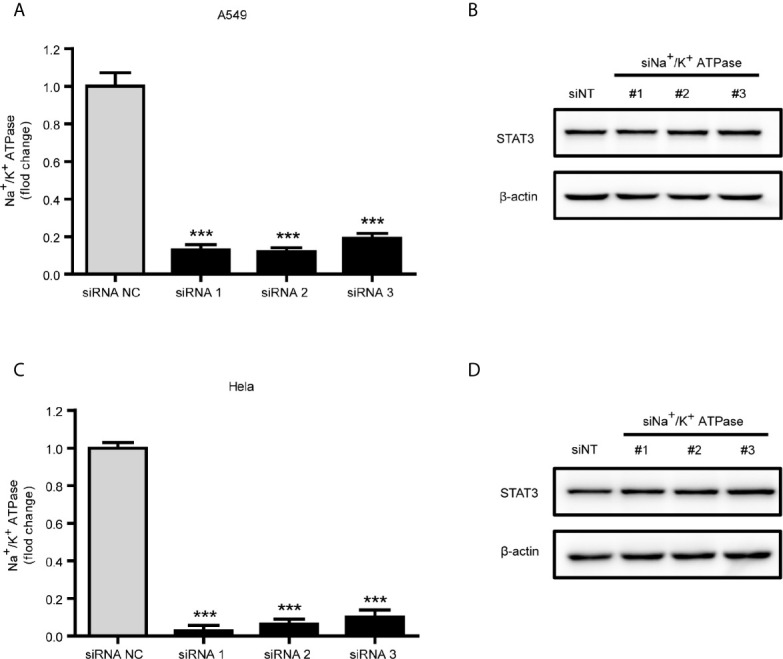
Downregulation of STAT3 by ouabain was independent of Na^+^/K^+^-ATPase. **(A, C)** Real-time qPCR verification of the Na^+^/K^+^-ATPase α1 subunit in A549 cells or Hela cells transfected with siRNAs specifically targeting the Na^+^/K^+^-ATPase α1 subunit. **(B, D)** Western blotting analysis of STAT3 in transfected cells from **(A)** or **(C)**. ^***^ *P* < 0.001 *versus* control group, n = 3.

Since the positive inotropic effects of ouabain are *via* binding and inhibition of the α1 subunit of Na^+^/K^+^-ATPase, we therefore further evaluated the role of Na^+^/K^+^-ATPase in ouabain-induced reduction of STAT3 expression. However, the reduction of STAT3 expression by ouabain treatment seemed to be independent of Na^+^/K^+^-ATPase α1 subunit, since knockdown of Na^+^/K^+^-ATPase α1 subunit by siRNA transfection did not affect the expression of STAT3 in A549 and Hela cells (**Figures 5A–D**).

### Ouabain Inhibited the Protein Synthesis of STAT3

**Figure 6:**
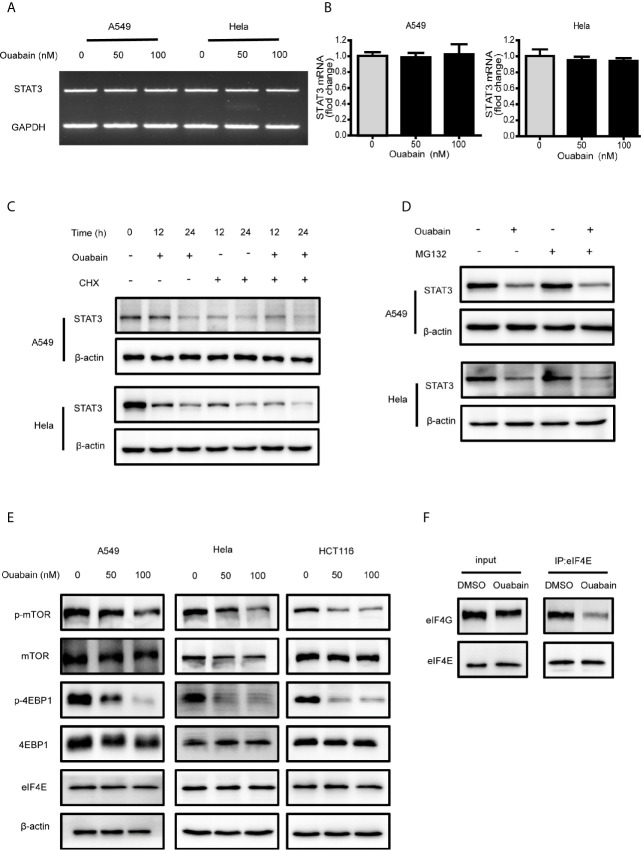
Ouabain inhibited the protein synthesis of STAT3. **(A, B)** A549 and Hela cells were exposed to ouabain for 24 hours, and STAT3 mRNA expression was determined by PCR. **(C, D)** A549 and Hela cells were treated with vehicle or ouabain in the presence or absence of cycloheximide (CHX) **(C)** or MG132 **(D)**, the expressions of STAT3 were analyzed by Western blotting. **(E)** A549, Hela and HCT116 cells were treated with different concentrations of ouabain for 24 hours, the expressions of p-mTOR, mTOR, p-4EBP1, 4EBP1 and eIF4E were analyzed by Western blotting. **(F)** Immunoprecipitation analysis of eIF4E and eIF4G in Hela cells treated with or without ouabain.

To elucidate the mechanism by which ouabain decreased STAT3 expression, we firstly performed semi-quantitative and quantitative real-time PCR to determine whether ouabain has a direct effect on the mRNA level of STAT3. As illustrated in **Figures 6A, B**, ouabain treatment did not alter the transcriptional levels of STAT3. To further determine whether ouabain-induced changes are associated with translational regulation, we treated cells with ouabain in the absence or presence of CHX, an inhibitor of protein synthesis. As shown in **Figure 6C**, the degradation rates of STAT3 were similar in both the CHX- treated and untreated cells, which indicates that ouabain-triggered reduction of STAT3 does not involve accelerating STAT3 protein degradation. Next, we used a proteasome inhibitor MG132 to verify this hypothesis. Our results showed that MG132 did not reverse the decreased STAT3 protein levels induced by ouabain (**Figure 6D**). Taken together, these data suggested that the reduction of STAT3 induced by ouabain might be the result of protein synthesis inhibition, rather than the promotion of protein degradation.

Protein synthesis initializes with the binding of eIF4F to the m^7^G cap at the 5’ end of eukaryotic mRNAs (20). The mTOR pathway could stimulate the phosphorylation of 4EBP1 and trigger the dissociation of 4EBP1 from eIF4E, allowing the association of eIF4E and eIF4G. To determine whether ouabain blocks protein synthesis at the level of initiation, we investigated the regulation of 4EBP1 and eIF4E. Western blot analysis revealed that ouabain treatment suppressed the activation of mTOR, as well as the phosphorylation of 4EBP1 in a dose-dependent manner (**Figure 6E**). In addition, immunoprecipitation showed the decreased association of eIF4E and eIF4G induced by ouabain treatment without affecting the protein expression of eIF4E and eIF4G (**Figure 6F**). We also observed that treatment with digoxin and lanatoside C downregulated the expression of STAT3 and phosphorylated 4EBP1 (**Figures S2C, D**). These results indicated that ouabain suppressed the STAT3 expression at the translational levels.

## Discussion

Cardiac glycosides are natural steroid compounds used for various cardiac diseases due to inhibition of Na^+^/K^+^-ATPase pumping activity. Actually, in recent years a growing body of research shows that the portfolio of diseases potentially treatable with cardiac glycosides is much broader. Classical drug digitoxin has been shown to block cytokine storm *via* suppressing the levels of the cytokines TNFα, GRO/KC, MIP2, MCP1 and IFN-γ, with implications for influenza and potentially for COVID-19 (21). Besides the well-known effect of ouabain on the cardiovascular system and blood pressure control, compelling evidences indicate that ouabain also acts as a regulator of various immune system functions including inflammation. Ouabain can modulate many inflammatory events such as cell migration, vascular permeability, cytokine production (16) and neuroinflammation. A previous report demonstrated that ouabain was capable to negatively modulate allergic airway inflammation induced by ovalbumin (22). However, more details about ouabain mechanism of action are needful on account of that ouabain has a pro- and anti-inflammatory effect, which mainly depends on its concentration and functional state of cells (23). Currently, cardiac glycosides are mostly studied as anticancer agents. The mechanisms of these anticancer effects may include induction of cell cycle arrest (24); inhibition of IL-8 production and of the NF-κB pathway (25); activation of the AMPK-Src signaling pathway (26) and suppression of HIF-1α protein synthesis (27). However, the efficacy of cardiac glycosides in cancer treatment remains controversial. A population-based case-control study reported that digoxin treatment was associated with an increased incidence of breast cancer (28). The conflict among these clinical observations suggests that more experimental evidences would contribute to solve the puzzle of anticancer mechanisms triggered by cardiac glycosides.

In present study, we verified the universal negative regulation of ouabain on human cervical cancer cells, non-small-cell lung cancer cells, colorectal carcinoma cells and pancreatic cancer cells. Nanomolar concentration of ouabain impaired cell viability, declined the colony formation ability and promoted apoptosis in a dose- and time-dependent manner. Further examination revealed the increase of intracellular ROS generation and DNA double-strand breaks induced by ouabain treatment in these cancer cell lines. In addition to its known actions, ouabain was capable to lower the expression of STAT3 and prevent the activation of STAT3 pathway signaling. Unexpectedly, specific knockdown of Na^+^/K^+^-ATPase α1 subunit by siRNA had no effect on STAT3 activation, indicating that Na^+^/K^+^-ATPase plays a negligible role in the ouabain-triggered reduction of STAT3. Furthermore, we provided experimental evidences that ouabain could decrease STAT3 expression at the translational levels.

In normal physiological conditions, the extent and duration of STAT3 activation are tightly controlled. However, abnormal upregulation of STAT3 activity would trigger malignant transformation and cancer progression by promoting oncogenic gene expression. Hence, abrogation of the STAT3 signaling pathway might represent an efficacious strategy for cancer prevention and therapy. Our results suggested that STAT3 might be one of the targets of ouabain, since the total expression and phosphorylation of STAT3 were greatly inhibited by ouabain. Accordingly, we found that the expression of anti-apoptotic proteins c-Myc, Survivin and Bcl-2, all of which have been reported to be regulated by STAT3, were downregulated following ouabain treatment. Consistent with our results, another two cardiac glycosides, oleandrin and odoroside A, have been reported to inhibit STAT3 activation in breast cancer cell line (29). Ouabain treatment could induce apoptosis through the decrease of intracellular K^+^ and increase of intracellular Na^+^ and Ca^2+^ (30–32), and the suppression of anti-apoptotic and pro-survival genes by STAT3 inactivation could synergistically promote these apoptosis-inducing effects. In addition, it has been shown digoxin and lanatoside C upregulated the ROS generation in hepatocellular carcinoma cells (33), and our data also revealed similar effects in ouabain-treated cancer cells. Ouabain was able to inhibit DNA damage repair directly by the Fanconi anemia/BRCA pathway (34) or indirectly by STAT3 inactivation (35–37), thus ouabain treatment could remarkably induce DNA double-strand breaks. Together, ouabain could exert its cytotoxic effects on cancer cells *via* multifaceted mechanisms.

To explore how ouabain suppressed STAT3 abundance, we measured the expression of STAT3 both in the mRNA and protein levels. Our data demonstrated that the reduction of STAT3 expression by ouabain occurred at the post-transcriptional levels. Further examination indicated that ouabain prohibited the protein synthesis of STAT3, possibly through the inhibition of mTOR/4EBP1 axis. The suppression of mTOR activity by ouabain led to hypo-phosphorylation of 4EBP1 which could bind and sequester eIF4E, thus prevented the formation of eIF4 complex and blocked translation initiation (38).

The Na^+^/K^+^-ATPase is the primary target of ouabain, and most of the biological actions of ouabain are dependent on the inhibition of Na^+^/K^+^ ion exchange and Na^+^/K^+^-ATPase-associated Src tyrosine kinase (26). However, transfection of siRNA targeting Na^+^/K^+^-ATPase α1 subunit did not alter the expression of STAT3 protein, implying a Na^+^/K^+^-ATPase-independent mechanism. Indeed, there are additional intracellular targets for cardiac glycosides. For example, ouabain-induced internalization of the Na^+^/K^+^-ATPase into endosomes has been reported in different kinds of cells (39, 40), and cardiac glycoside-induced endosomal recycling could further activate the degradation of other proteins, thus perturb cancer cell homeostasis (41). Cardiac glycosides could inhibit the L-type calcium current without the inhibition of Na^+^/K^+^-ATPase (42). In addition, ouabain and its metabolite, ouabagenin, are the ligands of estrogen receptor and the liver X receptor, respectively (43, 44). Recently, anti-coronaviral activity of ouabain has been reported to be Na^+^/K^+^-ATPase-independent proteolysis of Janus kinase 1 (45). Here, our data suggested that inactivation of STAT3 pathway signaling was not *via* the canonical Na^+^/K^+^-ATPase on the plasma membrane, and further investigation should be performed to fully illuminate the mechanism underlying the STAT3 inactivation by ouabain treatment in cancer cells.

In summary, although the pharmacological properties of ouabain are well-recognized, the mechanisms underlying potent antitumor activity of ouabain remain elusive. The current study provided the experimental evidence for STAT3 inhibition by ouabain in cancer cells. Regarding the compelling evidence for the oncogenic role of STAT3, abrogation of the STAT3 signaling pathway might play a fundamental role in the antitumor activity of ouabain. The current study might help to repurpose these cardiac glycosides as antitumor agents, alone or in combination with other therapeutic modalities.

## Data Availability Statement

The original contributions presented in the study are included in the article/**Supplementary Material**. Further inquiries can be directed to the corresponding author.

## Author Contributions

MZ and JD conceived and designed the experiments. JD, LJ, FC, and HH performed the experiments. JD and LJ analysed the data. MZ, JD, and LJ wrote the paper, reviewed and edited the manuscript. All authors contributed to the article and approved the submitted version.

## Funding

This work was supported by the National Natural Science Foundation of China (Grant No. 81903256 and 81673105; http://www.nsfc.gov.cn/) and the Natural Science Foundation of Guangdong Province (Grant No. 2019A1515010607 and 2020A151501664).

## Conflict of Interest

The authors declare that the research was conducted in the absence of any commercial or financial relationships that could be construed as a potential conflict of interest.
