# Supplementary material for: Cardiac Glycoside Ouabain Exerts Anticancer Activity via Downregulation of STAT3
Source: Front Oncol. 2021 Jun 30;11:684316. doi: 10.3389/fonc.2021.684316 (PMC8279743; doi:10.3389/fonc.2021.684316)
Supplement: Supplementary file 1 [file DataSheet_1.docx]

Supplementary Material

## SUPPLEMENTARY FIGURES

**
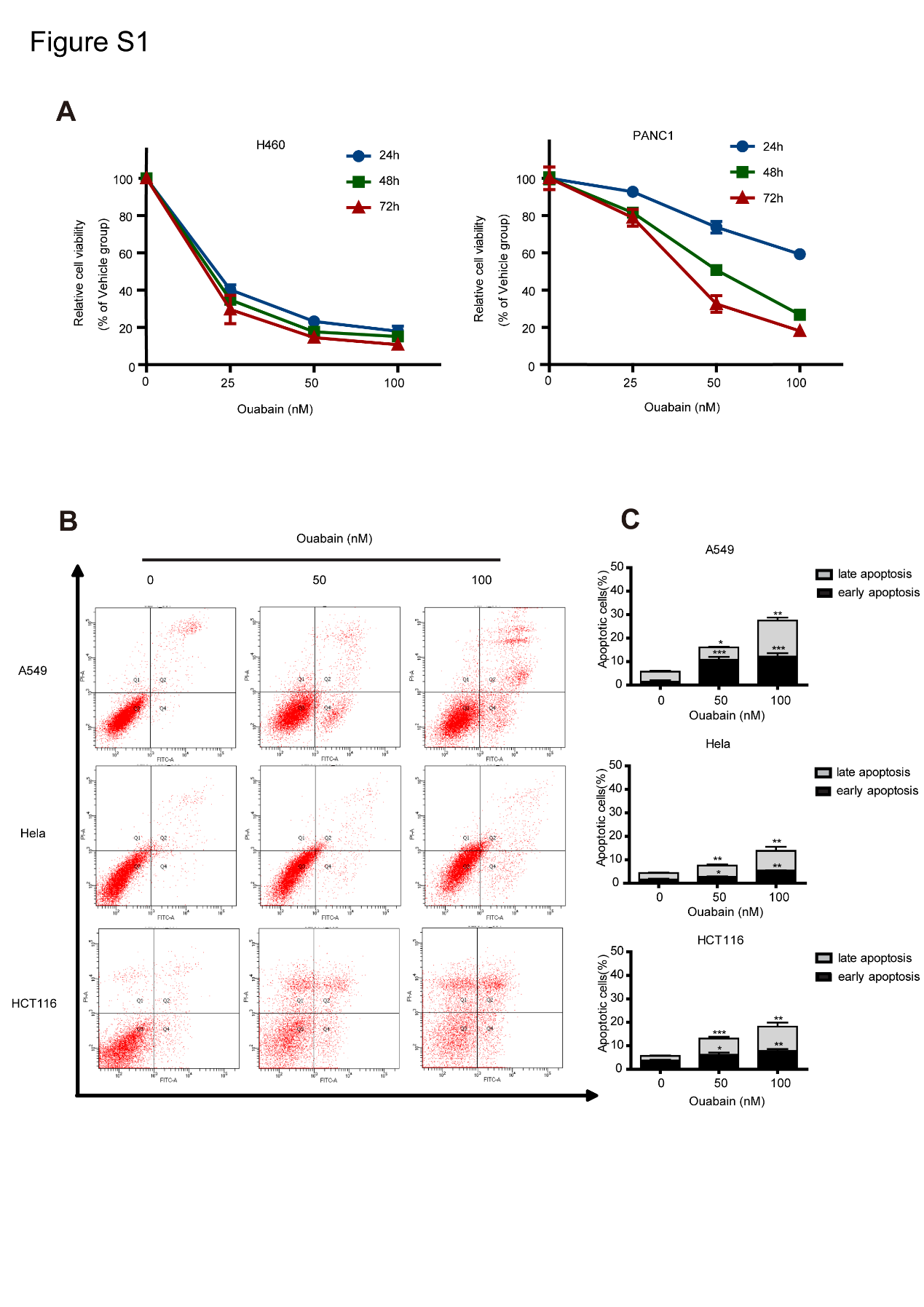
**

**Figure S1.** Ouabain decreased cell viability and induced apoptosis of cancer cells. **(A)** H460 and PANC1 cells were treated with ouabain (0-100 nM) for 24, 48, or 72 hours and cell viability was determined by CCK-8 assay. **(B) (C)** A549, Hela and HCT116 cells were treated with indicated dose of ouabain (0, 50, 100 nM) for 24 hours, apoptosis was tested by flow cytometric analysis with Annexin V-FITC/PI staining. ^*^, *P* < 0.05; ^**^, *P* < 0.01; ^***^, *P* < 0.001 versus control group, n = 3.

**
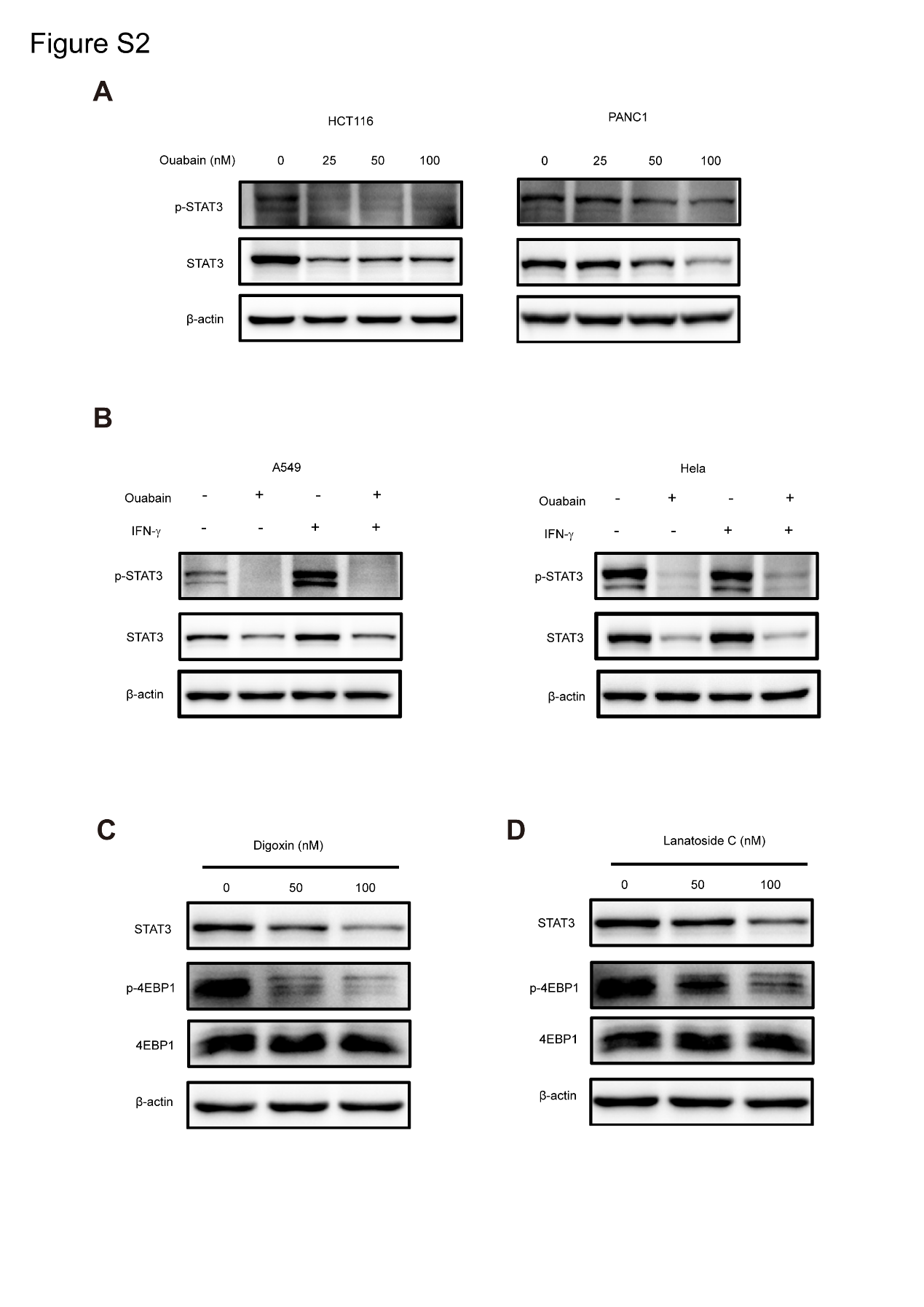
**

**Figure S2.** Ouabain decreased STAT3 activation and other cardiac glycoside (digoxin and lanatoside C) downregulated the expression of STAT3 and p-4EBP1. **(A)** H460 and PANC1 cells were incubated with ouabain for 24 hours, the expressions of p-STAT3 and STAT3 were analyzed by Western blotting. **(B)** Cytoplasmic extracts from A549 and Hela cells treated with vehicle, ouabain and IFN-γ for 24 hours were analyzed by Western blotting. **(C) (D)** Hela cells were treated with indicated doses of digoxin **(C)** and lanatoside C **(D)** for 24 hours, and expressions of STAT3 and p-4EBP1 were analyzed by Western blotting.
